# A Robust Rule-Based Framework for Stone Detection and Posterior Acoustic Shadow Localization in Abdominal Ultrasound

**DOI:** 10.3390/jimaging12040163

**Published:** 2026-04-09

**Authors:** Kyuseok Kim, Ji-Youn Kim

**Affiliations:** 1 Institute of Human Convergence Health Science, Gachon University, 191 Hambakmoero, Yeonsu-gu, Incheon 21936, Republic of Korea; kskim502@gachon.ac.kr; 2 Department of Dental Hygiene, Gachon University, 191 Hambakmoero, Yeonsu-gu, Incheon 21936, Republic of Korea

**Keywords:** automatic detection, rule-based image analysis, posterior acoustic shadowing, kidney and gallbladder stones, ultrasound image

## Abstract

Posterior acoustic shadowing is a fundamental physical phenomenon associated with calcified stones in ultrasound image, yet it has not been fully exploited in automated ultrasound analysis. This study aimed to develop an explainable, semi-automatic rule-based framework that explicitly incorporates posterior acoustic shadow characteristics for stone detection and localization in a clinically guided manner. A rule-based framework was designed to generate stone candidates using morphological enhancement and to evaluate them through local contrast analysis, posterior shadow region assessment, and shape-based penalties. A composite score integrating these features was used to rank candidates. The method was evaluated on 52 kidney stone and 66 gallbladder stone ultrasound images, stratified into three diagnostic confidence categories. Performance was assessed using an ablation study and centroid distance error measured in pixels relative to expert-defined references. In the 50–60% confidence group, the accuracy increased from 0.29 to 0.64 for kidney stones and from 0.30 to 0.60 for gallbladder stones when posterior shadow information was included. Centroid distance errors in the ≥80% confidence group were 1.26 ± 0.28 mm for kidney stones and 1.44 ± 0.91 mm for gallbladder stones. The proposed framework enhances diagnostic confidence by leveraging physically grounded posterior acoustic shadow analysis and provides a reproducible augmentation to conventional ultrasound-based stone assessment.

## 1. Introduction

Ultrasound imaging plays a central role in the non-invasive assessment of abdominal diseases, owing to its real-time imaging capability, widespread availability, absence of ionizing radiation, and low cost compared with computed tomography (CT) or magnetic resonance imaging (MRI) [1,2]. In clinical practice, ultrasound is frequently used for the initial evaluation of suspected kidney or gallbladder stones (i.e., urolithiasis and cholelithiasis), particularly in populations where repeated radiation exposure is a concern, and as a point-of-care tool in emergency and outpatient settings [3]. Despite its advantages, conventional B-mode ultrasound is known to have variable diagnostic accuracy for stones, with sensitivity and specificity dependent on stone size, location, and operator experience [4]. Thus, improving quantitative and automated methods to enhance ultrasonic stone detection remains an important research objective.

A fundamental imaging characteristic of urinary and biliary stones in diagnostic ultrasound is the presence of a hyperechoic focus accompanied by posterior acoustic shadowing, which arises from the strong acoustic impedance mismatch between calcified stone material and surrounding soft tissues [5]. When an incident ultrasound wave encounters a highly attenuating structure such as a stone, a substantial portion of the acoustic energy is either reflected or absorbed, resulting in a marked reduction in signal transmission beyond the stone. This physical phenomenon produces a distinct hypoechoic or anechoic region distal to the stone, commonly referred to as posterior acoustic shadowing. Importantly, this shadow is not merely an auxiliary visual cue but a direct manifestation of the stone’s intrinsic material properties, including composition, density, and effective cross-sectional area. Several clinical investigations have demonstrated that shadow-based measurements provide a more reliable surrogate for true stone size than direct measurements of the stone itself on B-mode ultrasound images. Because the bright stone surface is often subject to blooming artifacts, speckle noise, and partial volume effects, direct delineation of stone boundaries can be inconsistent, particularly for small stones or those located in anatomically complex regions [6,7]. In contrast, the posterior shadow integrates cumulative attenuation effects and is less sensitive to local intensity fluctuations. Retrospective studies comparing ultrasound measurements with the CT have reported that stone width estimated from shadow dimensions exhibits smaller mean absolute error and improved correlation with CT-derived stone size than conventional ultrasound-based stone measurements [2,8]. These findings indicate that posterior acoustic shadowing encodes clinically meaningful information that more faithfully reflects the physical extent of the stone.

Despite its well-recognized clinical significance, posterior acoustic shadowing remains largely underutilized in automated ultrasound analysis pipelines. Most existing computer-aided diagnosis (CAD) approaches for stone detection rely primarily on local intensity characteristics, such as hyperechogenicity, edge sharpness, or blob-like morphology [9,10]. While such strategies are effective in detecting bright regions, they are inherently vulnerable to false positives caused by non-stone structures, including vessel walls, fascial interfaces, bowel gas, and specular reflections. Moreover, intensity-based features alone do not adequately capture the downstream acoustic consequences of a stone, which are often more stable and discriminative than the stone signal itself [11]. Advanced quantitative texture analysis methods, including gray-level co-occurrence matrix (GLCM), gray-level run length matrix (GLRLM), and related statistical descriptors, have been successfully applied in medical imaging modalities to characterize tissue heterogeneity and pathological patterns [1,12,13]. However, in conventional ultrasound-based stone analysis, such techniques have predominantly been applied to the stone region or surrounding parenchyma, rather than to the posterior shadow itself. The primary reason for this omission lies in the lack of robust methods to automatically and reproducibly localize the shadow region, particularly in the presence of fan-shaped imaging geometry, depth-dependent attenuation, and anatomical variability. As a result, the posterior shadow, despite its diagnostic relevance, has remained difficult to integrate into automated frameworks, creating a methodological gap between clinical knowledge and algorithmic implementation.

The need for improved automatic detection and localization of stones extends beyond methodological refinement and carries substantial clinical implications. In renal stone disease, accurate identification and localization of stones influence key management decisions, including whether conservative observation is appropriate or whether intervention is warranted. Stone size and morphology are strongly associated with the likelihood of spontaneous passage, the risk of obstruction, and the choice of treatment modality. Inaccurate or inconsistent stone localization on ultrasound may therefore lead to delayed diagnosis, unnecessary follow-up imaging, or inappropriate clinical decision-making [14,15]. A robust ultrasound-based method that leverages posterior acoustic shadow information could enhance diagnostic confidence while reducing reliance on CT, particularly in populations requiring repeated imaging. Similarly, in gallbladder stone disease, reliable detection is essential for determining surgical indications and preventing complications such as cholecystitis or biliary obstruction. While CT provides high diagnostic accuracy, its routine use is limited by ionizing radiation exposure and cost, making ultrasound the preferred first-line modality in most clinical settings. However, operator dependency and image variability continue to limit ultrasound performance in certain cases. By incorporating physically grounded shadow-based criteria into an automated framework, ultrasound examinations can be augmented with objective and reproducible analysis, potentially improving diagnostic consistency across operators and institutions. Consequently, the development of automated methods that explicitly exploit posterior acoustic shadowing represents not only a technical advancement but also a clinically motivated step toward safer, more reliable, and more accessible stone evaluation.

In this study, we propose an automated framework that systematically identifies stone candidates on ultrasound images and employs posterior acoustic shadow analysis. Unlike conventional methods that emphasize brightness or geometric features of hotspots alone, our method prioritizes shadow onset estimation to enhance diagnostic performance. The framework is validated on clinical datasets of kidney and gallbladder stones categorized by diagnostic confidence. The proposed approach has the potential to serve as an explainable, reproducible augmentation to routine ultrasound examination protocols, bridging the gap between clinical expertise and algorithmic diagnosis.

## 2. Materials and Methods

### 2.1. Abdominal Ultrasound Image Acquisition

Ultrasound examinations were retrospectively collected in accordance with the approval of the Institutional Review Board (IRB) of Gachon University (Approval No. 1044396-202411-HR-185-01; its waiver was approved on 20 November 2024), which waived the requirement for informed consent due to the retrospective nature of the study.

All ultrasound images were acquired using a LOGIQ S7 R3 Expert diagnostic ultrasound system (GE Healthcare, Chicago, IL, USA) equipped with a wide-band convex transducer (C1–5-D, bandwidth 2–5 MHz, field of view 69°). The examinations were performed using a standardized abdominal preset. To minimize variability across acquisitions, imaging parameters including center frequency, imaging depth, focal zone placement, overall gain, time gain compensation (TGC), dynamic range, and post-processing options such as speckle reduction and spatial compounding were kept consistent throughout all examinations. For more detailed ultrasound acquisition and imaging parameters, see Supplementary S1.

Ultrasound data were collected between 2022 and 2023 from patients with suspected kidney or gallbladder stones. A total of 52 ultrasound images of kidney stones and 66 ultrasound images of gallbladder stones were included in the study. For gallbladder examinations, patients were scanned after a fasting period according to routine clinical protocols to ensure adequate gallbladder distension. Images were acquired in the supine position, with additional oblique or lateral positions when necessary to obtain optimal visualization of the stone and its posterior acoustic shadow. For kidney examinations, both longitudinal and transverse planes were assessed, and frames in which the echogenic focus and the associated posterior acoustic shadow were simultaneously visible were selected for analysis. All ultrasound images were independently reviewed by two experienced physicians and three certified sonographers, each with more than 10 years of clinical experience in abdominal ultrasonography. The reviewers confirmed the presence of stones and evaluated the characteristics of posterior acoustic shadowing. Posterior acoustic shadowing is a well-recognized sonographic phenomenon associated with calcified structures such as kidney and gallbladder stones. In clinical practice, the presence, strength, and continuity of posterior shadowing are routinely used by experienced examiners as indirect indicators of stone composition and diagnostic certainty. Following previously reported clinical grading concepts that classify posterior shadowing as weak, moderate, or strong, this study adopted a shadow-strength-based diagnostic confidence framework to stratify ultrasound images into three probability categories.

Rather than representing statistically computed probabilities, the assigned probability ranges (50–60%, 60–80%, and ≥80%) reflect expert-defined diagnostic confidence levels, grounded in visual assessment of posterior acoustic shadow characteristics. This approach aligns with prior retrospective ultrasound studies in which diagnostic likelihood was inferred from qualitative and semi-quantitative shadowing patterns rather than model-based probability outputs. The diagnostic confidence categories were defined based on the morphological and intensity characteristics of posterior acoustic shadowing immediately distal to the echogenic focus suspected to represent a stone. The operational definitions used in this study are summarized as follows [16]:

- Low confidence (50–60%)—Weak shadowing

Images in this category exhibited faint, discontinuous, or poorly defined posterior attenuation. The shadow was typically short in axial extent, partially obscured by speckle noise, or inconsistent across adjacent scan lines. In some cases, the shadow was only appreciable under specific gain or viewing conditions, limiting diagnostic certainty.

- Intermediate confidence (60–80%)—Moderate shadowing

Moderate shadowing was characterized by a clearly identifiable posterior attenuation zone that was continuous but limited in depth or lateral extent. The shadow was darker than the surrounding tissue and spatially aligned with the echogenic focus, yet its boundaries were not sharply delineated or were partially interrupted by background structures.

- High confidence (≥80%)—Strong shadowing

Strong shadowing was defined as a well-demarcated, continuous, and homogeneous hypoechoic region extending distally from the echogenic focus. The shadow persisted over a substantial axial depth, exhibited clear lateral boundaries comparable to or wider than the stone width, and demonstrated high contrast relative to adjacent tissue. These features were considered highly characteristic of true calcified stones.

This qualitative grading scheme corresponds conceptually to previously reported weak–moderate–strong shadow classifications and was mapped to the three diagnostic probability ranges to facilitate structured analysis. The shadow-based diagnostic confidence labels established in this study were subsequently used as reference categories for evaluating the proposed automated stone detection and posterior shadow extraction framework. The expected probability of diagnosis is defined as the empirical proportion of correctly detected cases within each expert-defined diagnostic confidence category. It is computed as the ratio of the number of correctly detected cases to the total number of cases in the corresponding group. This metric does not represent a model-derived probabilistic output, but rather reflects the observed detection performance within each confidence level.

Importantly, the classification reflects clinically grounded diagnostic confidence, rather than algorithmic prediction probability, allowing the proposed method to be interpreted in the context of real-world ultrasound practice. By anchoring automated analysis to expert-defined posterior shadow characteristics, the proposed framework aims to bridge the gap between traditional qualitative ultrasound interpretation and objective computational image analysis.

### 2.2. Proposed Framework

**Figure 1:**
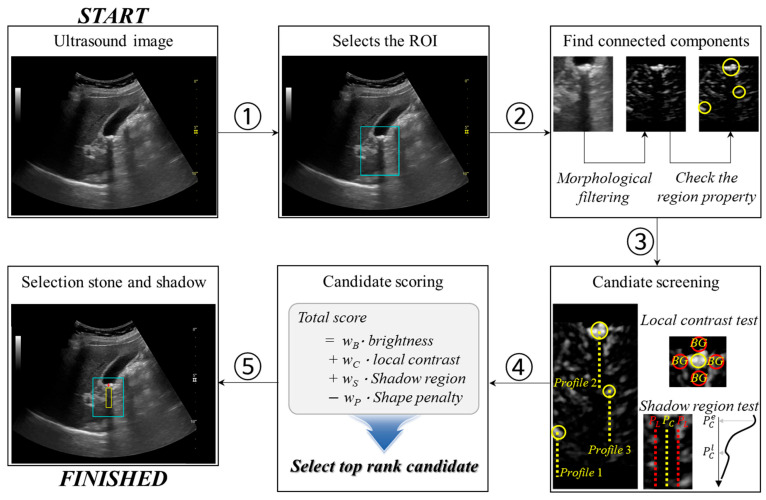
A schematic overview of the proposed automatic stone detection and posterior acoustic shadow extraction framework in ultrasound images.

Figure 1 shows that the proposed framework automatically detects stone candidates and extracts the posterior acoustic shadow region beneath the selected stone.

Initially, the region of interest (ROI) was manually defined prior to algorithm execution, following routine clinical ultrasound interpretation practice (①). Rather than attempting fully automated organ localization, the proposed framework assumes that a clinician or trained operator initially designates a suspected region containing the kidney or gallbladder stone. This design reflects standard clinical ultrasound practice, where examiners actively identify suspicious anatomical regions during real-time scanning and focus subsequent interpretation on these areas. Rather than performing fully automated whole-image analysis, the framework is designed to operate as a decision-support tool that evaluates candidate regions within a clinically selected ROI. This approach reduces the influence of irrelevant background structures and allows the algorithm to focus on physically meaningful acoustic features, particularly posterior acoustic shadowing. In addition, the use of a manually defined ROI enables controlled validation of the proposed shadow-based analysis framework by minimizing variability associated with automated organ localization. This is particularly important in ultrasound imaging, where anatomical appearance, field of view, and acquisition conditions can vary substantially across patients and scanning protocols. The ROI was drawn as a rectangular region encompassing the visually suspected stone location and its immediate downstream region where posterior acoustic shadowing is expected to occur. All subsequent processing steps including candidate generation, screening, scoring, and posterior shadow extraction were performed exclusively within this manually defined ROI. The ROI image is converted to double precision and normalized to [0,1] using min–max normalization. Speckle noise is reduced using median filtering with a small kernel (e.g., 3 × 3 pixels), which improves robustness of subsequent morphological enhancement and thresholding. To emphasize locally hyperechoic stone-like structures while suppressing slowly varying background intensity, morphological top-hat filtering is applied to the masked ROI image (②). This is implemented using *imtophat* built-in MATLAB R2025b version (MathWorks, Natick, MA, USA) function with a disk-shaped structuring element, where the radius controls the scale of enhanced bright objects. Morphological top-hat filtering was performed using a disk-shaped structuring element with a radius of 5 pixels, which was selected to enhance locally bright stone-like structures while suppressing background variations. Stone candidates are initially defined as pixels exceeding a high-percentile threshold in the top-hat enhanced image (e.g., the 99th percentile), generating a binary candidate map [17,18]. Small isolated detections are removed using *bwareaopen* built-in MATLAB function with a minimum area constraint. Small isolated candidate regions were removed using a minimum area threshold of 20 pixels to suppress noise-induced detections. Regional maxima detection is used to enforce peak-like candidate selection and further suppress broad bright regions. The remaining candidate pixels are grouped into connected components using *bwconncomp* built-in MATLAB function. For each connected component, region-level descriptors are computed using *regionprops* built-in MATLAB R2025b version (MathWorks, Natick, MA, USA) function, including area, mean intensity, centroid location, bounding box, eccentricity, solidity, and major or minor axis lengths ($L_{max}$ and $L_{min}$). Here, the shape penalty is defined as a weighted combination of three region-based morphological descriptors extracted using connected-component analysis:

(1) $$shape\;penalty=\alpha \cdot eccentricity+\beta \cdot \max\left(0,1-solidity\right)+\gamma \cdot \max\left(0,\frac{L_{max}}{L_{min}}\right).$$

The first term, eccentricity, quantifies the degree to which a candidate resembles a line-like structure rather than a compact object. Values approaching unity indicate highly elongated shapes, which are more characteristic of boundaries or reverberation artifacts than true stones. The second term penalizes low solidity, reflecting fragmented or concave shapes commonly produced by speckle noise or partially connected reflections. The third term evaluates anisotropy via the major-to-minor axis ratio and imposes a penalty only when elongation exceeds a predefined tolerance, thereby avoiding excessive suppression of mildly elliptical stones. α = 0.4, β = 0.3, and γ = 0.3 are weighting coefficients empirically selected to balance sensitivity and specificity. To reduce computation in subsequent screening, only the top *K* candidates (e.g., *K* ≤ 5) are retained based on a brightness such as

(2) $$brightness=\mu \sqrt{A},$$

where μ and A denote the mean intensity and area of candidate, respectively. Candidates are then analyzed independently.

Then, we perform two screenings of the candidates: (1) local contrast test and (2) shadow region test (③). In local contrast test, a surrounding ring region is defined by expanding the bounding box in each direction. The mean intensity inside the candidate ring ($\bar{I_{s}}$) and the mean intensity in the surrounding ring ($\bar{I_{r}}$) are computed:

(3) $$local\;contrast=\bar{I_{s}}-\bar{I_{r}}.$$

Here, candidates with $local\;contrast<\tau_{LC}$ are rejected. $\tau_{LC}$ is set to 300, empirically. In implementation, binary masks are constructed for the candidate box and ring region, and the ring mask excludes the candidate box and is restricted to valid pixels. For local contrast evaluation, the bounding box of each candidate was expanded by 5 pixels in all directions to define the surrounding ring region used for intensity comparison. In shadow region test, a posterior shadow score is computed by comparing intensity profiles below the candidate to confirm downstream acoustic consequences. Let y denote a row just below the candidate bottom. The central profile is obtained by averaging intensity across a narrow band centered at the candidate centroid:

(4) $$P_{C}(y)={mean}_{x\in \left[x_{c}-h,x_{c}+h\right]}I(y,x),$$

where $x_{c}$ is x-axis of center profile and h is a fixed window depth, which is defined as a vertical analysis segment. And the lateral reference profile ($P_{L}(y)$) is obtained by averaging left and right bands offset laterally by a gap. A shadow region is then computed as

(5) $$shadow\;region={mean}_{y}\left(P_{L}\left(y\right)-P_{C}\left(y\right)\right)\;+\rho (median(P_{C}^{e})-median(P_{C}^{l})),$$

where $P_{C}^{e}$ and $P_{C}^{l}$ are the value of the profile where the value drops most rapidly after starting at the stone site and the profile where the value rises most rapidly, respectively. The posterior shadow profile was computed using a central analysis band with a width of 5 pixels centered at the candidate centroid. The vertical analysis depth was set to 30 pixels, and lateral reference profiles were defined with an offset of 10 pixels from the central axis. ρ is balancing parameter between two terms, and it is set to 0.5, empirically. Thus, higher values of the lateral–center intensity difference indicate selective beam attenuation caused by a stone, rather than uniformly reduced echogenicity, which explains why this term increases rather than vanishes in true stone cases. Candidates with $shadow\;region<\tau_{SR}$ are rejected. $\tau_{SR}$ is set to 100, empirically.

Remaining candidates are ranked using a composite score combining brightness, local contrast, posterior shadow strength, and a shape penalty (④):

(6) $$Total\;score=w_{B}\cdot brightness+w_{C}\cdot local\;contrast+w_{S}\cdot shadow\;region-w_{P}\cdot shadow\;penalty,$$

where $w_{B}$, $w_{C}$, $w_{S}$, and $w_{P}$ are weight factors for each term. The composite score was computed as a weighted combination of brightness, local contrast, posterior shadow strength, and shape penalty, with weighting factors empirically set to $w_{B}$ = 0.35, $w_{C}$ = 0.25, $w_{S}$ = 0.25, and $w_{P}$ = 0.15, respectively. Finally, using the selected candidate centroid and the estimated shadow onset depth, the predicted center position of the stone is represented as an indicator, the posterior shadow ROI is extracted as a rectangular region directly beneath the stone (⑤).

### 2.3. Quantitative Metrics

For each ultrasound image, the algorithm outputs a single predicted stone location. A case was considered correctly detected when the predicted point fell within the expert-annotated suspected stone region. Accuracy was computed as follows [19]:

(7) $$Accuracy=\frac{N_{correct}}{N_{total}},$$

where $N_{correct}$ is the number of images in which the predicted stone point satisfied the acceptance rule, and $N_{total}$ is the number of images in that group.

To quantitatively assess the accuracy of stone localization, we evaluated the spatial discrepancy between the automatically predicted stone position and the reference annotation provided by clinical experts. For each ultrasound image, an expert manually identified the suspected stone region and marked two end points corresponding to the extremities of the stone along its visually estimated long axis. These two end points were connected to form a reference straight line representing the principal axis of the stone. Let the two expert-defined end points be denoted as $A=(x_{a},y_{a})$, $B=(x_{b},y_{b})$, which define a straight line in the image plane. The automatically detected stone location was represented by the centroid of the predicted stone candidate, denoted as $O=(x_{o},y_{o})$. The localization error was defined as the perpendicular (orthogonal) distance from the predicted centroid to the reference line. The reference line was expressed in the general form ax+by+c=0, which the cofficients a, b, and c were derived from the two end points A and B. The centroid distance error d was then computed as

(8) $$d=\frac{\left|ax_{o}+by_{o}+c\right|}{\sqrt{a^{2}+b^{2}}}.$$

which corresponds to the shortest Euclidean distance between the predicted stone location and the expert-defined stone axis. The distance was measured in mm and reflects the degree of alignment between the automatic detection result and the expert’s estimation of the stone position. The centroid distance error between the reference value and the predicted value is an evaluation method used in a lot of papers [20,21] and can be used as a basis for evaluating the accuracy of the proposed method. To assess interobserver reliability, two independent observers (each with more than 10 years of clinical experience in abdominal ultrasonography) annotated the stone axis by selecting two endpoints for each image. For each annotation, the two endpoints were used to derive representative parameters, including the center coordinates and the axis length. Interobserver agreement was quantified using the intraclass correlation coefficient (ICC) for continuous variables, including center coordinates and axis length. ICC values greater than 0.75 were interpreted as good agreement, and values greater than 0.90 as excellent agreement.

### 2.4. Statistical Analysis

All quantitative results are presented as mean ± standard deviation. The 95% confidence intervals (CIs) were calculated using the t-distribution. Comparisons between groups were descriptive due to limited sample size. Statistical analyses were performed using MATLAB software R2025b version (MathWorks, Natick, MA, USA).

## 3. Results and Discussion

**Figure 2:**
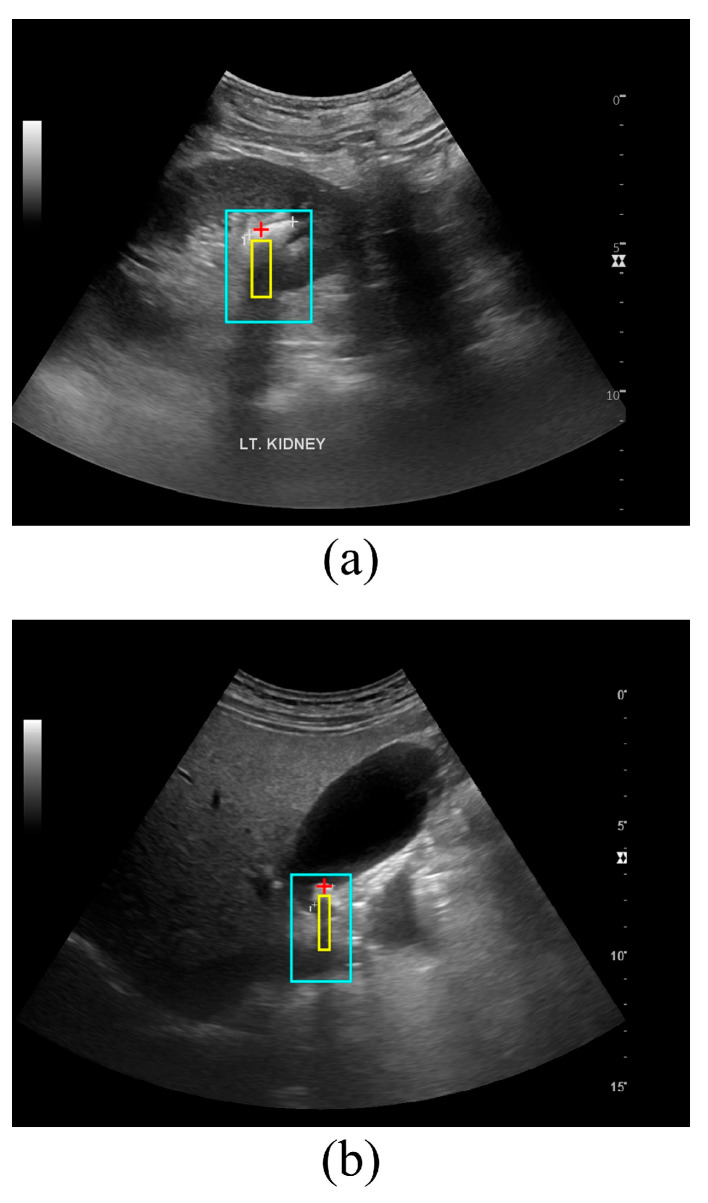
Representative results of automatic stone localization and posterior acoustic shadow detection using the proposed method: (**a**) kidney and (**b**) gallbladder.

Figure 2 presents examples of stone localization and posterior acoustic shadow detection obtained using the proposed method in ultrasound images of (a) the kidney and (b) gallbladder. As shown in Figure 2a, the algorithm accurately localized the renal stone by identifying a high-echogenic focus, followed by the detection of a vertically elongated low-intensity region posterior to the stone, corresponding to the acoustic shadow. The detected shadow region was spatially aligned with the estimated stone center, indicating consistent geometric correspondence between the stone and its posterior attenuation pattern. Similarly, Figure 2b illustrates the application of the proposed method to a gallbladder ultrasound image. Despite differences in surrounding anatomy and background echogenicity compared with the kidney, the method successfully identified the gallstone and its associated posterior acoustic shadow. The shadow region extended inferiorly from the detected stone location, reflecting typical ultrasound attenuation behavior caused by highly attenuating calcified structures. Across both anatomical sites, the proposed method demonstrated robust performance in simultaneously detecting stone locations and their posterior acoustic shadows, suggesting that the underlying detection strategy is not limited to a specific organ. These qualitative results support the general applicability of the proposed approach to ultrasound-based stone detection in anatomically and acoustically diverse environments.

Table 1 summarizes the ablation study results of the proposed total score in Equation (6) for kidney and gallbladder stone detection. The diagnostic performance was evaluated by progressively incorporating feature components including brightness, local contrast, posterior acoustic shadow region, and a penalty term, and analyzing their impact on the accuracy. Accuracy was computed for each organ and confidence group using the predefined acceptance criterion, allowing group-wise evaluation of detection performance. The number of cases in each confidence group was as follows: for the kidney dataset, *n* = 15 (≥80%), *n* = 23 (60–80%), and *n* = 14 (50–60%); for the gallbladder dataset, *n* = 35 (≥80%), *n* = 21 (60–80%), and *n* = 10 (50–60%). For the kidney dataset, the use of brightness alone resulted in moderate diagnostic probabilities, with values of 0.73, 0.52, and 0.29 for the ≥80%, 60–80%, and 50–60% probability ranges, respectively. When local contrast was added, the expected probability increased consistently across all ranges, indicating that local intensity variation provides complementary information beyond absolute brightness. Further inclusion of the posterior acoustic shadow region led to a notable improvement, particularly in the higher probability ranges, suggesting that shadow-related features contribute substantially to increasing diagnostic confidence. The full model incorporating brightness, local contrast, shadow region, and the penalty term achieved the highest performance, with expected probabilities of 0.93, 0.86, and 0.64, respectively. A similar trend was observed in the gallbladder dataset. Brightness-based scoring alone yielded limited performance, especially in the 50–60% probability range. The addition of local contrast improved the expected probability, while incorporating the posterior acoustic shadow region further enhanced performance across all probability ranges. The complete formulation of the total score, including the penalty term, achieved the highest expected probabilities, demonstrating consistent gains compared to partial feature combinations. Overall, the ablation results indicate that while brightness and local contrast contribute to baseline discrimination, the inclusion of posterior acoustic shadow information plays a critical role in increasing diagnostic confidence. The additional penalty term further refines the total score by suppressing less reliable detections, leading to improved performance in both kidney and gallbladder datasets. These findings suggest that each component of the proposed total score contributes additively and consistently across different anatomical sites. Due to the limited number of samples within each confidence subgroup, formal statistical hypothesis testing was not performed. Therefore, the reported improvements should be interpreted as descriptive trends rather than statistically validated differences.

**Figure 3:**
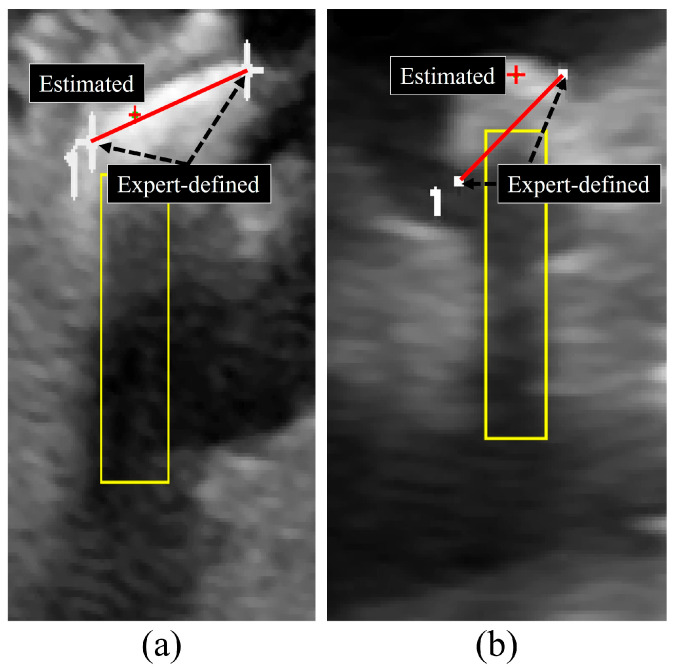
Illustration of the quantitative evaluation scheme for centroid distance error: (**a**) kidney and (**b**) gallbladder. For each suspected stone, a reference line is defined by two expert-annotated endpoints representing the major axis of the stone.

Figure 3 illustrates representative examples of the quantitative evaluation scheme used to assess the agreement between expert annotations and the stone location predicted by the proposed method. For each suspected stone, an expert manually defined a reference line segment by selecting two endpoints corresponding to the perceived major axis of the stone. This expert-defined line served as a geometric reference for evaluating the spatial accuracy of the automatically estimated stone location. Interobserver reliability analysis demonstrated high consistency between the two observers. The ICC values for the stone center coordinates were 0.88 for the x-axis and 0.91 for the y-axis, while the ICC for axis length was 0.87, indicating good to excellent agreement. These results suggest that the expert-defined reference annotations were stable and reproducible, supporting the validity of the quantitative evaluation framework used in this study. Figure 3a,b show enlarged views of the cyan-boxed regions, highlighting the local anatomical context in which the stone was suspected and annotated.

Table 2 shows the centroid distance error, measured in pixels, between the expert-defined reference and the stone location predicted by the proposed method for kidney and gallbladder ultrasound images. The centroid distance error was computed as the shortest Euclidean distance, in pixel units, between the predicted stone center and the expert-defined reference line. Results are reported as mean ± standard deviation together with 95% CI. To improve clinical interpretability, pixel-based localization errors were additionally converted into physical distance (mm) using an empirically derived spatial resolution of about 0.35 mm/pixel based on the ultrasound system configuration. Expressing localization error in millimeters improves clinical interpretability and facilitates comparison with physical stone dimensions reported in prior studies. For the kidney dataset, the centroid distance error was 1.26 ± 0.28 mm (95% CI: 1.12–1.41 mm) in the ≥80% expected probability range. The error increased to 2.10 ± 0.67 mm (95% CI: 1.91–2.46 mm) in the 60–80% range and further to 6.51 ± 6.16 mm (95% CI: 3.40–9.85 mm) in the 50–60% range, indicating increased spatial dispersion of predicted stone locations at higher expected probability thresholds. For the gallbladder dataset, the centroid distance error was 1.44 ± 0.91 mm (95% CI: 1.06–1.67 mm) in the ≥80% range, increasing to 2.42 ± 0.70 mm (95% CI: 2.11–2.72 mm) in the 60–80% range, and reaching 4.03 ± 0.64 mm (95% CI: 3.63–4.42 pixels) in the 50–60% range. Compared with the kidney dataset, the gallbladder results exhibited a more gradual increase in centroid distance error across probability ranges. In the kidney dataset, the standard deviation of the centroid distance error in the 50–60% expected probability group was relatively large and approached the corresponding mean value. This increased variability is likely attributable to limitations in image reliability rather than deficiencies in the proposed algorithm. In particular, renal stones often exhibit indistinct posterior acoustic shadows, and the definition of the stone long axis may be inherently unstable in cases with weak or fragmented shadowing, leading to increased dispersion in expert-defined references. In contrast, the gallbladder dataset showed a more gradual increase in standard deviation across expected probability ranges compared with the kidney dataset. This difference can be explained by anatomical and acoustic characteristics of gallstones, which typically present with clearer boundaries and more spatially consistent posterior acoustic shadows. As a result, both expert annotation and automated localization tend to be more stable in gallbladder ultrasound images. These observations underscore the importance of confidence-stratified evaluation in ultrasound-based stone detection. By analyzing localization error within different expected probability ranges, the evaluation framework allows separation of algorithmic behavior from image-dependent uncertainty, providing a more nuanced interpretation of performance under varying levels of diagnostic confidence.

**Figure 4:**
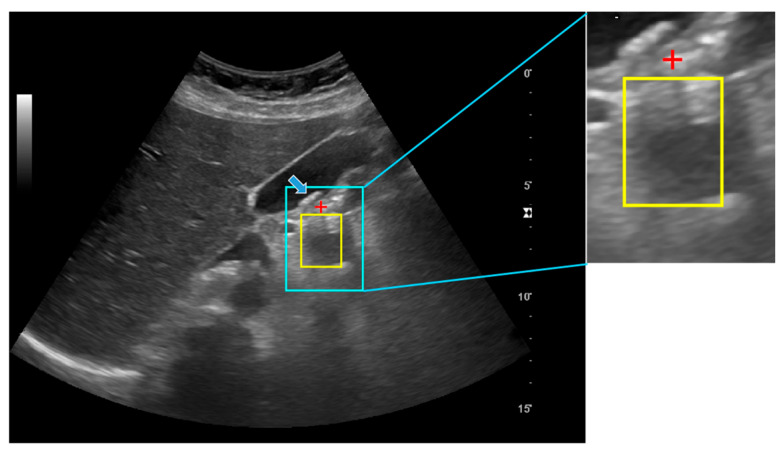
Fail case of automatic stone localization and posterior acoustic shadow detection using the proposed method and its enlarged image in gallbladder ultrasound image.

The proposed method was effective in detecting stone locations and associated posterior acoustic shadow regions; however, several limitations remain. To provide a more structured interpretation of these limitations, failure cases were qualitatively categorized into four representative types. First, weak or discontinuous posterior acoustic shadowing reduced the reliability of shadow-based verification, particularly in low-confidence cases. Second, small stones often produced insufficient attenuation and only limited downstream shadow formation, making them difficult to distinguish from surrounding hyperechoic structures. Third, high levels of speckle noise interfered with both candidate generation and posterior shadow profile estimation. Fourth, confounding echogenic structures, such as vessel walls, fascial interfaces, or specular reflections, occasionally mimicked stone-like appearance and resulted in false-positive detections. Among these categories, sensitivity to ultrasound image reliability and acoustic physics was a particularly important limitation. The visibility and geometric consistency of posterior shadowing can vary substantially with acquisition settings and patient-specific factors, which may propagate to unstable definition of expert long-axis references and increased localization dispersion even when the algorithmic logic is unchanged. Figure 4 presents a representative failure case belonging to the weak- or ambiguous-shadow category. In this example, the arrows indicate the stone locations expected by experts, whereas the red crosses and yellow boxes denote the stone positions and posterior shadow regions predicted by the proposed method. Although additional subgroup-specific quantitative experiments were beyond the scope of the current study, this qualitative error analysis suggests several directions for improvement. Future work could incorporate an explicit shadow-confidence or visibility score to gate or weight predictions, acquisition-aware normalization (e.g., compensation for gain and spatial compounding effects), and multi-cue fusion strategies that combine hyperechoic stone appearance with posterior shadow structure, rather than relying on a single acoustic cue in low-confidence cases.

Another limitation is that the quantitative localization error is reported in pixels, which improves internal consistency but can reduce interpretability across scanners and protocols because ultrasound systems may exhibit device- and setup-dependent spatial sampling and measurement behaviors. Pixel-domain evaluation is useful when physical calibration metadata are unavailable or unreliable; however, it makes it harder to compare outcomes across institutions or to translate improvements into clinically intuitive magnitudes [22]. A practical solution is a dual-reporting strategy, which involves maintaining pixel-based metrics for strict algorithmic comparison while additionally providing calibrated physical units when feasible using standardized QA or calibration procedures (e.g., phantom-based distance accuracy checks) [23] and scanner metadata such as alongside sensitivity analyses, showing how conclusions change with scaling assumptions.

Third, a limitation arises from the fact that the evaluation reference itself can be unstable, especially when experts must define a stone’s long axis or a representative reference line under ambiguous visual evidence. In ultrasound, interobserver differences can occur even in standard biliary examinations, and kidney stone assessment is known to exhibit nontrivial observer variability, meaning that a portion of the reported centroid distance error may reflect reference variability rather than purely algorithmic error [24,25]. This issue can become more pronounced when the dataset is small, because a few difficult cases can disproportionately shape summary statistics within each probability bin. We expect that the problem will be sufficiently overcome if we secure more datasets and conduct evaluations through further research.

Finally, a key limitation of this study is that the dataset was acquired from a single institution using a single ultrasound system. Although this controlled setting allowed consistent evaluation of the proposed framework, it may limit generalizability across different scanners, acquisition protocols, and patient populations. Future work will include multi-center and cross-institutional validation using heterogeneous datasets to assess robustness under varying clinical conditions. In addition, external validation using independent datasets acquired from different ultrasound systems was not performed due to limited availability of annotated posterior shadow data. This will be addressed in future studies to confirm the robustness of the proposed framework.

Future work will extend the proposed shadow-ROI-based verification and scoring into a multi-cue stone detection framework that explicitly combines complementary ultrasound evidence to improve accuracy in challenging cases (e.g., weak or ambiguous posterior shadowing, strong speckle, or confounding echogenic structures). In particular, we expect that integrating the proposed score with color doppler twinkling artifact features will increase sensitivity for small or subtle stones while maintaining specificity by cross-checking candidates against shadow-consistent texture signatures [26,27]. Prior clinical and technical studies have shown that twinkling artifact can serve as an additional diagnostic cue for urinary and biliary stones, and that its performance depends on acquisition and machine settings, suggesting strong synergy with a physics-motivated verification score like ours that can stabilize decision-making across heterogeneous appearances. In parallel, we will develop a reliability-aware deployment pipeline so that the combined system can generalize to broader scanners, protocols, and institutions as the dataset grows. Unlike deep learning-based methods such as convolutional neural network, U-Net, or YOLO, the proposed framework emphasizes interpretability and physical consistency [28,29]. Future work will include comparative evaluation with data-driven approaches when sufficiently large annotated datasets become available. A key goal is to couple the proposed scoring and verification stage with explicit uncertainty estimation and robustness strategies to support confidence-aware triage (e.g., automated acceptance for high-confidence cases and human review when uncertainty is high), which is especially important when ultrasound evidence is intrinsically ambiguous [30]. In addition, because labeled stone datasets are often limited and institution-specific, we will investigate data-efficient learning (e.g., few-shot or meta-learning or related strategies) to reduce annotation burden while preserving performance under domain shift [31]. These directions are supported by recent medical imaging literature emphasizing structural uncertainty estimation for segmentation reliability and systematic evidence that few-shot learning can mitigate data scarcity in medical imaging tasks.

From a practical perspective, the proposed framework is composed of computationally efficient and deterministic image processing steps, making it suitable for real-time or near-real-time implementation. The absence of iterative training procedures or large-scale neural networks enables low computational overhead and facilitates integration into existing ultrasound workstations. In a clinical setting, the proposed method can function as a decision-support tool by providing objective and reproducible assessment of posterior acoustic shadow characteristics within clinician-selected regions of interest. This may help reduce operator dependency and improve diagnostic consistency across users and institutions. Furthermore, the explainable nature of the framework allows clinicians to interpret the underlying decision logic, which is an important requirement for clinical adoption.

## 4. Conclusions

In this study, we presented a robust, rule-based framework for automatic stone detection and posterior acoustic shadow localization in abdominal ultrasound images, with a particular focus on kidney and gallbladder stones. Unlike conventional intensity-driven approaches, the proposed method explicitly exploits posterior acoustic shadow characteristics, which are a direct physical consequence of stone–tissue acoustic interactions and a clinically meaningful indicator routinely used by experienced sonographers. By integrating brightness, local contrast, shadow-region analysis, and shape-based penalties into a unified scoring scheme, the framework demonstrated consistent improvements in expected diagnostic probability across confidence strata in both organs. Quantitative evaluation using a centroid distance error metric further showed that the proposed method achieves reasonable spatial agreement with expert annotations while enabling a nuanced interpretation of performance through confidence-stratified analysis.

## Figures and Tables

**Table 1:** Ablation study results showing the accuracy, defined as the proportion of correctly detected cases within each confidence category.

**Kidney**	**Accuracy**		
	***n*** ** = 15** **(≥80%)**	***n*** ** = 23** **(60–80%)**	***n*** ** = 14** **(50–60%)**
brightness	0.73	0.52	0.29
brightness, local contrast	0.86	0.65	0.43
brightness, local contrast, shadow region	0.93	0.78	0.57
brightness, local contrast, shadow region and penalty	0.93	0.86	0.64
**Gallbladder**	**Accuracy**		
	***n*** ** = 35** **(≥80%)**	***n*** ** = 21** **(60–80%)**	***n*** ** = 10** **(50–60%)**
brightness	0.80	0.52	0.30
brightness, local contrast	0.86	0.67	0.50
brightness, local contrast, shadow region	0.91	0.81	0.60
brightness, local contrast, shadow region and penalty	0.94	0.90	0.60

**Table 2:** Centroid distance error (mm) between expert-defined references and automatically predicted stone locations.

**Kidney**	**Expected Probability of Diagnosis**		
	**≥80%**	**60–80%**	**50–60%**
Centroid distance error	1.26 ± 0.28 [1.12–1.41]	2.10 ± 0.67 [1.91–2.46]	6.51 ± 6.16 [3.40–9.85]
**Gallbladder**	**Expected Probability of Diagnosis**		
	**≥80%**	**60–80%**	**50–60%**
Centroid distance error	1.44 ± 0.91 [1.06–1.67]	2.42 ± 0.70 [2.11–2.72]	4.03 ± 0.64 [3.63–4.42]

## Data Availability

The raw data supporting the conclusions of this article will be made available by the authors upon request.

## Supplementary Materials

The following supporting information can be downloaded at https://www.mdpi.com/article/10.3390/jimaging12040163/s1, Supplementary S1. Ultrasound acquisition and imaging parameters.

## References

[1] Dai J.C., Dunmire B., Sternberg K.M., Liu Z., Larson T., Thiel J., Chang H.C., Harper J.D., Bailey M.R., Sorensen M.D. (2018). Retrospective comparison of measured stone size and posterior acoustic shadow width in clinical ultrasound images. World J. Urol..

[2] Tzou D.T., Usawachintachit M., Taguchi K., Chi T. (2017). Ultrasound use in urinary stones: Adapting old technology for a modern-day disease. J. Endourol..

[3] Smith-Bindman R., Aubin C., Bailitz J., Bengiamin R.N., Camargo C.A., Corbo J., Dean A.J., Goldstein R.B., Griffey R.T., Jay G.D. (2014). Ultrasonography versus computed tomography for suspected nephrolithiasis. N. Engl. J. Med..

[4] Brisbane W., Bailey M.R., Sorensen M.D. (2016). An overview of kidney stone imaging techniques. Nat. Rev. Urol..

[5] Baad M., Lu Z.F., Reiser I., Paushter D. (2017). Clinical significance of US artifacts. RadioGraphics.

[6] Ganesan V., De S., Greene D., Torricelli F.C.M., Monga M. (2017). Accuracy of ultrasonography for renal stone detection and size determination: Is it good enough for management decisions?. BJU Int..

[7] Dunmire B., Harper J.D., Cunitz B.W., Lee F.C., Hsi R., Liu Z., Bailey M.R., Sorensen M.D. (2016). Use of the acoustic shadow width to determine kidney stone size with ultrasound. J. Urol..

[8] Dai J., Dunmire B., Liu Z., Sternberg K.M., Bailey M.R., Harper J.D., Sorensen M.D. (2018). Measurement of posterior acoustic stone shadow on ultrasound is a learnable skill for inexperienced users to improve accuracy of stone sizing. J. Endourol..

[9] Lian J., Ma Y., Ma Y., Shi B., Liu J., Yang Z., Guo Y. (2017). Automatic gallbladder and gallstone regions segmentation in ultrasound image. Int. J. Comput. Assist. Radiol. Surg..

[10] Nicolau C., Claudon M., Derchi L.E., Adam E.J., Nielsen M.B., Mostbeck G., Owens C.M., Nyhsen C., Yarmenitis S. (2015). Imaging patients with renal colic-consider ultrasound first. Insights Imaging.

[11] May P.C., Haider Y., Dunmire B., Cunitz B.W., Thiel J., Liu Z., Bruce M., Bailey M.R., Sorensen M.D., Harper J.D. (2016). Stone-mode ultrasound for determining renal stone size. J. Endourol..

[12] Aerts H.J.W.L., Velazquez E.R., Leijenaar R.T.H., Parmar C., Grossmann P., Carvalho S., Bussink J., Monshouwer R., Haibe-Kains B., Rietveld D. (2014). Decoding tumour phenotype by noninvasive imaging using a quantitative radiomics approach. Nat. Commun..

[13] Lambin P., Rios-Velazquez E., Leijenaar R., Carvalho S., van Stiphout R.G.P.M., Granton P., Zegers C.M.L., Gillies R., Boellard R., Dekker A. (2012). Radiomics: Extracting more information from medical images using advanced feature analysis. Eur. J. Cancer.

[14] Jendeberg J., Geijer H., Alshamari M., Cierzniak B., Lidén M. (2017). Size matters: The width and location of ureteral stone accurately predict the chance of spontaneous passage. Eur. Radiol..

[15] Ordon M., Andonian S., Blew B., Schuler T., Chew B., Pace K.T. (2015). CUA guideline: Management of ureteral calculi. Can. Urol. Assoc. J..

[16] Hanafi M.Q., Fakhrizadeh A., Jaafaezadeh E. (2019). An investigation into the clinical accuracy of twinkling artifacts in patients with urolithiasis smaller than 5 mm in comparison with computed tomography scanning. J. Family Med. Prim. Care.

[17] Xi T., Yuan L., Sun Q. (2022). A combined approach to infrared small-target detection with the alternating direction method of multipliers and an improved top-hat transformation. Sensors.

[18] Zeng M., Li J., Peng Z. (2006). The design of top-hat morphological filter and application to infrared target detection. Infrared Phys. Technol..

[19] Sokolova M., Lapalme G. (2009). A systematic analysis of performance measures for classification tasks. Inf. Process. Manag..

[20] Qureshi A., Lim S., Suh S.Y., Mutawak B., Chitnis P.V., Demer J.L., Wei Q. (2023). Deep-learning-based segmentation of extraocular muscles from magnetic resonance images. Bioengineering.

[21] Jin C., Udupa J.K., Zhao L., Tong Y., Odhner D., Pednekar G., Nag S., Lewis S., Poole N., Mannikeri S. (2022). Object recognition in medical images via anatomy-guided deep learning. Med. Image Anal..

[22] Goldstein A. (2000). Errors in ultrasound digital image distance measurements. Ultrasound Med. Biol..

[23] Fabiszewska E., Pasicz K., Grabska I., Skrzyński W., Ślusarczyk-Kacprzyk W., Bulski W. (2017). Evaluation of imaging parameters of ultrasound scanners: Baseline for future testing. Pol. J. Radiol..

[24] Grantcharov T.P., Rasti Z., Rossen B., Kristiansen V.B., Rosenberg J. (2002). Interobserver agreement in ultrasound examination of the biliary tract. Acta Radiol..

[25] Festi D., Lalloni L., Taroni F., Barbara L., Menotti A., Ricci G. (1989). Inter and intea-observer variation in ultrasonographic detection of gallstones: The multicenter Italian study on epidemiology of cholelithiasis (M.I.COL.). Eur. J. Epidemiol..

[26] Masch W.R., Cohan R.H., Ellis J.H., Dillman J.R., Rubin J.M., Davenport M.S. (2016). Clinical effectiveness of prospectively reported sonographic twinkling artifact for the diagnosis of renal calculus in patients without known urolithiasis. AJR Am. J. Roentgenol..

[27] Sorensen M.D., Harper J.D., His R.S., Shah A.R., Dighe M.K., Carter S.J., Moshiri M., Paun M., Lu W., Bailey M.R. (2013). B-mode ultrasound versus color doppler twinkling artifact in detecting kidney stones. J. Endourol..

[28] Rudin C. (2019). Stop explaining black box machine learning models for high stakes decisions and use interpretable models instead. Nat. Mach. Intell..

[29] Litjens G., Kooi T., Bejnordi B.E., Setio A.A.A., Ciompi F., Ghafoorian M., van der Laak J.A.W.M., van Ginneken B., Sánchez C.I. (2017). A survey on deep learning in medical image analysis. Med. Image Anal..

[30] Yang B., Zhang X., Zhang H., Li S., Higashita R., Liu J. (2025). Structural uncertainty estimation for medical image segmentation. Med. Image Anal..

[31] Pachetti E., Colantonio S. (2024). A systematic review of few-shot learning in medical image. Artif. Intell. Med..

