# Supplementary material for: A Robust Rule-Based Framework for Stone Detection and Posterior Acoustic Shadow Localization in Abdominal Ultrasound
Source: J Imaging. 2026 Apr 9;12(4):163. doi: 10.3390/jimaging12040163 (PMC13117089; doi:10.3390/jimaging12040163)
Supplement: Supplementary file 1 [file jimaging-12-00163-s001.zip › jimaging-4221452-supplementary.pdf]

Supplementary S1. Ultrasound acquisition and imaging parameters

All ultrasound images used in this study were acquired using a LOGIQ S7 R3 Expert diagnostic ultrasound system (GE Healthcare, Chicago, IL, USA) equipped with a wide-band convex transducer (C1–5-D) with a bandwidth of 2–5 MHz. The center frequency was set to approximately 3.5 MHz. The field of view was 69°, and the imaging depth ranged from approximately 10 to 14 cm depending on patient anatomy.

All examinations were performed using a standardized abdominal preset to ensure consistency across acquisitions. To minimize variability, key imaging parameters including focal zone placement, overall gain, time gain compensation (TGC), and dynamic range were maintained as consistently as possible throughout all examinations. The overall gain was set to approximately 65–70%, and the dynamic range was approximately 60 dB. A consistent TGC profile was applied across all scans.

Post-processing settings included moderate speckle reduction and enabled spatial compounding, with low persistence to preserve temporal resolution. The beamforming mode and line density were maintained as system default settings and were not explicitly modified. Ultrasound data were acquired as cine loops at approximately 30 frames per second, and representative frames were selected for analysis. All images were exported as grayscale ultrasound images and processed as normalized intensity data. Prior to analysis, images were converted to double precision and normalized using min–max scaling, and speckle noise was reduced using median filtering. The spatial resolution of the images was estimated to be approximately 0.35 mm/pixel, which was used to convert localization error from pixel units to physical distance (mm) for improved clinical interpretability. All imaging parameters were kept as consistent as possible to reduce acquisition-related variability and ensure reproducibility of the proposed framework.
